# How do mass shootings shape the social media discourse on guns in the US Congress? Causal discovery and topic modeling

**DOI:** 10.1371/journal.pgph.0005493

**Published:** 2025-12-17

**Authors:** Dmytro Bukhanevych, Rayan Succar, Maurizio Porfiri

**Affiliations:** 1 Center for Urban Science and Progress, Tandon School of Engineering, New York University, Brooklyn, New York, United States of America; 2 Department of Mechanical and Aerospace Engineering, Tandon School of Engineering, New York University, Brooklyn, New York, United States of America; 3 Department of Biomedical Engineering, Tandon School of Engineering, New York University, Brooklyn, New York, United States of America; 4 Department of Civil and Urban Engineering, Tandon School of Engineering, New York University, Brooklyn, New York, United States of America; PLOS: Public Library of Science, UNITED STATES OF AMERICA

## Abstract

Social media platforms have become a key tool for politicians to signal their policy positions and communicate about issues that are salient to them and their constituency. One such issue is gun violence. Grounded in framing and issue-attention cycle theories, this paper analyzes the response of members of the United States (US) Congress to mass shootings on social media. We analyzed 785,881 gun-related tweets from members of the 117^th^ US Congress on X (formerly Twitter) between January 2021 and January 2023. We used logistic regression to model the main effects, implemented the PCMCI+ algorithm for causal discovery, and applied latent Dirichlet allocation topic modeling to evaluate the substantive differences between gun-related tweets from the two parties. Higher fatality counts were positively correlated with the probability of gun-related tweets by Congress members (OR=1.13, 95% CI=[1.12, 1.15], *p* < 0.001). A causal link was detected between mass shootings and subsequent legislators’ activity on X (*ρ*=0.122, *p*=0.001). Democrats were more likely to tweet about guns following mass shootings than Republicans (OR=3.60, 95% CI=[3.03, 4.28], *p* < 0.001), with qualitative differences in tweet substance between parties (community, families, victims, and mass shootings themselves are recurrent topics for Democrats, while Second Amendment rights and crime are frequent for Republicans). The paper suggests that while mass shootings elevate the level of discussion on guns in Congress, they trigger different reactions depending on party affiliation. Congress members tend to focus on topics aligned with party issues, likely reducing the opportunity for policy-making alignment.

## Author summary

Guns are one of the most divisive political issues in the US, consistently sparking intense public debate. While mass shootings are universally condemned by policymakers, how exactly they shape their gun discourse is presently unknown. By examining tweets from members of the 117^th^ US Congress, we confirmed the general intuition that mass shootings significantly increase the probability of policymakers posting about gun-related issues. The volume and content of those tweets differ by party, potentially amplifying political polarization and hindering a constructive legislative discourse towards addressing the public health epidemic of gun violence.

## 1 Introduction

Shocking events often elicit strong and immediate [1] public reaction, and they trigger calls to respond to crises [2]. Politicians react via public statements and, more recently, tweets to signal their position on a variety of topics, especially in relation to public health [3,4] as we have seen during the recent COVID-19 pandemic [5,6]. This mode of communication almost resembles “the rundown of the nightly news by capturing those issues which are most salient [for members of the US Congress]” [7]. X (formerly Twitter) is an important tool for communication for members of Congress, providing politicians an avenue for voicing positions that could be barred from traditional news and amplifying party positions [7,8]. The statements made on social media can be viewed as more direct and frank exemplars of a politician’s position, to a point where voters perceive politicians as more honest on social media, compared to news interviews [9].

Mass shootings are part of the broader public health epidemic of gun violence in the US [10]. They are recurrent [11], dramatic events [12] that draw the attention of the general public and media to gun violence and gun control [13,14]. The discourse in the aftermath of a mass shooting often brings back similar responses by politicians, such as offering “thoughts and prayers” to affected families and communities and engaging in divisive discussions around gun-related topics [15]. Research on the response of Congress members in the wake of the Parkland shooting [16] pointed at critical differences in the responses between Democrats and Republicans. There is a general lack of systematic studies on the discourse by Congress members on social media around gun-related topics in the aftermath of these events, thereby challenging a complete understanding of how the US political leadership communicates around gun violence, an intensifying public health crisis [17–19]. In democratic countries, the public is highly responsive to cues from political leaders [20–22]; therefore, an improved knowledge about how the US Congress communicates gun-related opinions is a crucial public health topic for the country. With the political ascension of X’s owner, Elon Musk, communication on X by politicians is a particularly timely topic to investigate.

We approach the analysis of social media discourse from two linked theoretical frameworks. The first one is the issue-attention cycle – a theory describing the patterns of public attention to an issue [2,23]. The second one is the framing theory, which posits that a specific framing of reality, such as a politician’s tweet about a mass shooting, will highlight certain aspects of the story and omit others. This may cause the audience to have different reactions to the same event [17,24].

**Fig 1:**
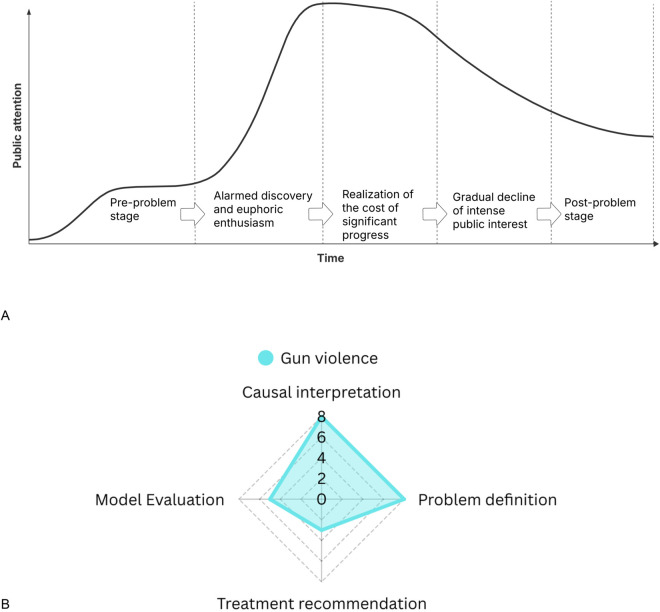
Illustration of the theoretical frameworks underpinning the study. (A) issue-attention cycle and (B) framing theory. (A) Time series of how public attention changes over time through five stages: pre-problem stage, alarmed discovery and euphoric enthusiasm, realization of the cost of significant progress, gradual decline of intense public interest, and post-problem stage. Figure adapted from Downs [2]. (B) The scoring across four framing dimensions (problem definition, causal interpretation, treatment recommendation, and model evaluation) was evaluated based on our personal judgement of the issue and how we tackled it in this study. Figure adapted from Entman [24].

The issue-attention cycle, introduced by Downs [2], provides a lens for understanding the dynamics of public and political focus on social problems such as mass shootings. According to this theory, public attention to major social issues follows a predictable cycle that includes five stages (Fig 1A): (1) the pre-problem stage, (2) alarmed discovery and euphoric enthusiasm, (3) realization of the cost of significant progress, (4) gradual decline of intense public interest, and (5) the post-problem stage. In the context of mass shootings, these events typically propel the issue of gun violence into stage 2 (alarmed discovery), prompting immediate public outrage and political reaction. However, as media coverage wanes and legislative inaction persists, the issue recedes from public attention until the next major event.

Framing theory complements this approach by pointing at how political actors may construct messages to influence public perception. As Entman [24] articulates, framing involves selecting and emphasizing certain aspects of perceived reality in order to promote a particular interpretation (Fig 1B). In the case of mass shootings, politicians may frame these events in terms of policy failure, personal tragedy, or constitutional rights, depending on their political ideology, audience, and strategic objectives. Framing is particularly salient on platforms like X, where messages are brief, direct, and often emotionally charged. Partisan divergence in framing serves both as a reflection of policy priorities and a mechanism for shaping public opinion in line with ideological commitments.

In contrast with existing studies that focused on the response on social media of the US Congress to a specific mass shooting, we propose a longitudinal investigation over a two-year window to help pinpoint when and how members of the 117^th^ Congress discuss gun-related topics on X. Based on prior research that identified the number of fatalities as a salient metric of general public’s interest [25] and media coverage [26], and the overwhelming evidence about political polarization in the US [16,27,28], we specifically test and find evidence to support the following hypotheses: (i) there is a positive relationship between the lethality of a mass shooting (measured in terms of the number of victims killed) and the likelihood of members of Congress posting a gun-related tweet, (ii) the political affiliation of members of Congress (Democratic versus Republican) is associated with differences in the relationship between mass shooting severity and the volume of gun-related tweets, and (iii) there is a thematic difference between Democrats and Republicans in the way they discuss gun-related issues.

## 2 Materials and methods

### 2.1 Study design

We conducted a retrospective time series analysis using X activity data spanning the period between January 2, 2021 and January 3, 2023 (the 117^th^ Congress). The study population comprised 513 members of the 117^th^ Congress. Political affiliation was coded as one for Democrats and zero for Republicans. Using logistic regression, we tested the effect of the number of fatalities of mass shootings on the probability of gun-related tweets posted by members of Congress. We controlled for political affiliation, location, and time delays. We further controlled for some social and demographic determinants, like income inequality (Gini coefficient), and residential segregation, which are commonly considered for gun violence analysis [29]. We used race/ethnicity of the members of Congress as an additional control variable, based on previous work pointing to a role of race/ethnicity on one’s stance on gun rights debate [30]. We did not consider the race/ethnicity of the victims and the suspects/perpetrators of the shootings, as this data is not reported by the Gun Violence Archive due to multiple reasons, such as inaccuracies in police reporting and personal healthcare information protection laws. This study utilized data from X users who had consented to disclose their content under the platform’s privacy policy. Institutional ethical approval was not required, as we did not interact directly with human subjects while analyzing internet activity. Moreover, user identities were not used, and all information remained anonymized [31].

### 2.2 Data sources

**Tweets.** Overall, 785,881 tweets were collected from the social media account handles from the US Congress Legislators data repository [32], maintained through manual edits by volunteers from GovTrack, ProPublica, MapLight, FiveThirtyEight, and others. We utilized a scraper for social networking services [33] (which is often used to scrape X [34]) to collect publicly available tweets posted by official members’ accounts, in compliance with the platform’s terms and services at the time of data collection in August 2023. A total of 513 members were included in the study, of them 262 were Democrats (51%) and 251 Republicans (49%). Independent members and those who did not have an X account (28 out of 541 members in total) were excluded from the analysis.

**Mass shootings.** In total, 1,338 mass shooting incidents (as defined by the Gun Violence Archive - events in which four or more people were shot – murdered or injured – excluding the shooter) were registered during the 117^th^ Congress, according to the Gun Violence Archive database[35].

**Gun-related tweets.** In total, 12,274 tweets by members of the 117^th^ Congress contained gun-related keywords. We used keywords from Benton [36] to select general gun-related tweets: gun, second amendment, 2nd Amendment, and firearm.

**Social & demographic data.** We used one of the most common measures of inequality – income inequality (the state-level Gini coefficient using 2023 Census data [37]) to control for social disparity. Additionally, state-level residential segregation (index of dissimilarity [38]) was used to control for another salient social determinant of exposure to gun violence [29]. We used data from Pew Research Center [39] and Congress.gov [40] to manually compile the ethnic background of the members of Congress.

### 2.3 Logistic regression

**Fig 2:**
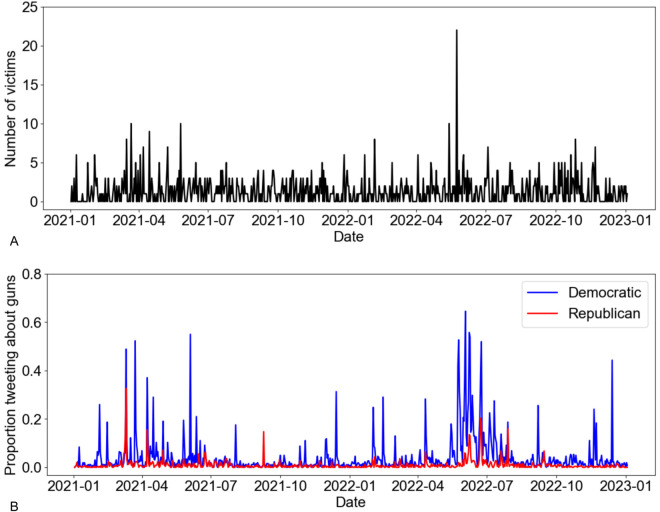
Time series of gun-related discourse and incidents in the US Congress. (A) The highest number of fatalities of a single mass shooting event in the US from January 3, 2021 to January 3, 2023. If multiple mass shootings occurred, the one with the highest death toll was selected. (B) Proportion of Democratic and Republican members of the 117^th^ Congress tweeting about guns.

We used mixed-effects logistic regression analysis to address the first hypothesis *(i) there is a positive relationship between the lethality of a mass shooting (measured in terms of the number of victims killed) and the likelihood of members of Congress posting a gun-related tweet*. Such an approach allows us to explore how mass shootings affect Congress members’ gun-related rhetoric online. The main goal was to understand if more severe shootings would make Congress members more likely to tweet about gun issues and if this probability varied between Democrats and Republicans. Fig 2 illustrates the time series of the variables of interest. The dependent variable was coded as one if a member of Congress tweeted at least one gun-related tweet on a given day and as zero otherwise. Hence, the regression was performed on 513×732=375,516 data points (513 accounts and 732 days, see Table 1.).

**Table 1:** Descriptive statistics for variables used in analyses, United States, January 3, 2021 to January 3, 2023. Sample size n=375,516. Means are presented with standard errors in parentheses. Percentages represent the proportion of observations with the characteristic. Location variable indicates whether the most lethal shooting event on the day occurred in the Congress member’s state. Race/ethnicity percentages represent unique members of Congress by demographic category.

Variable	% or Mean (SE)
**Dependent variable**	
Gun-related tweets	2.58% (0.03%)
**Focal independent variables**	
Number of fatalities	1.33 (0.003)
Location (in-state most lethal shooting)	2.54% (0.03%)
Political affiliation (Democrats)	51.07% (0.08%)
**Control variable**	
Total fatalities	1.89 (0.004)
Income inequality (GINI coefficient)	0.476 (0.0000)
Residential segregation index	62.64 (0.012)
Race/ethnicity (White)	75.24%
Race/ethnicity (Black)	10.33%
Race/ethnicity (Hispanic)	8.77%
Race/ethnicity (Asian American)	3.70%
Race/ethnicity (Multiracial)	1.17%
Race/ethnicity (Native American)	0.78%

For the first and simplest model (Model 1), we included four independent variables: the number of fatalities of a mass shooting on the given day, the political affiliation of the tweet author (Democrat or Republican), the Gini coefficient for the state where the shooting took place, and the residential segregation index. If there were multiple mass shootings recorded on a given day, the shooting with the highest death toll was selected. If multiple states experienced the same highest death toll on the same day, we averaged the Gini coefficient and the residential segregation index of the different states, respectively. To control for possible spatial dependencies, we introduced a binary variable–location–that was coded as one if the deadliest shooting happened in the same state as represented by the member of Congress and zero otherwise (or if no shooting occurred). The last variable we controlled for is the race/ethnicity of members of Congress. As noted previously [41,42], social and demographic factors play a crucial role in understanding mass shootings. Elements such as income inequality and personal identity within broader structural contexts influence not only the prevalence of mass shootings but also public attitudes toward gun policy. These dynamics can shape how salient a shooting becomes in public discourse and increase the pressure on legislators to respond.

In a second model (Model 2), we accounted for temporal autoregression by introducing three additional independent variables into the logistic regression: the one-day history of the dependent variables representing whether a Congress member posted a gun-related tweet, the number of fatalities of a single shooting in the previous day, and the location value in the previous day.

Finally, as a robustness check for the primary variable, we replicated the logistic regression analysis from Model 2, while substituting one independent variable (and its one-day history). Specifically, we replaced the highest single-day death toll across all mass shootings with the total number of casualties on that day (Model 3).

The tweets of each member of Congress over time were treated as the repeated measure. All *p* values were from two-sided tests, and results were deemed statistically significant at *α* = 0.050. Logistic regression analysis was performed in Python using Pymer4 library [43] and R using lme4 library [44]. We used the Akaike information criterion (AIC) and the Bayesian information criterion (BIC) tests to compare the goodness of fit [45]. These model selection criteria serve as a tool for comparing the goodness of fit for different models: the lower the values, the better the goodness of fit. Thus, we selected the model that minimized AIC and BIC values [46].

### 2.4 Time series analysis

In order to address hypothesis *(ii) the political affiliation of members of Congress (Democratic versus Republican) is associated with differences in the relationship between mass shooting severity and the volume of gun-related tweets* and obtain a deeper insight into the causal structure of our dynamical variables, we used the PCMCI+ (Peter-Clark Momentary Conditional Independence) algorithm with partial correlation as the conditional independence test. PCMCI+ is a sophisticated causal discovery algorithm that is designed to identify dependencies in multivariate time series data, extending the classical PC algorithm to handle the temporal dynamics of complex systems. Our three temporal variables were: the number of fatalities of a single mass shooting on a given day (Fig 2A), the proportion of Republican members of Congress (with X accounts) tweeting about guns on a given day, and the proportion of Democratic members of Congress (with X accounts) tweeting about guns on a given day (Fig 2B).

We used the Tigramite Python library [47] for causal discovery with the PCMCI+ algorithm. At first, PCMCI+ assumes a fully connected causal graph, and then it iteratively removes links between variables that are conditionally independent to refine the network structure. Once the skeleton of dependencies is established, links are oriented by temporal precedence, following the principle that past events influence future ones. Instantaneous relationships are then identified using deterministic orientation rules based on principles of causal inference pioneered by Pearl [48]. To capture the temporal dynamics of the mass shooting event and subsequent discussion on X, we set the minimum time lag at $\tau_{\min}=0$ and the maximum at $\tau_{\max}=7$.

### 2.5 Latent Dirichlet allocation

Finally, to test for possible qualitative differences of gun-related tweets by members of both parties of Congress, we employed latent Dirichlet allocation (LDA) for topic modeling of the tweets that were posted in the aftermath of mass shootings. This addresses hypothesis *(iii) there is a thematic difference between Democrats and Republicans in the way they discuss gun-related issues.* LDA is widely used to infer distinctive topics and discover patterns in a text corpus [49], including for tweets [50,51]. Specifically, for the LDA, we included all gun-related tweets that were tweeted on a day that had at least one mass shooting or at least one mass shooting the day before. This selection was informed by the causal relationships identified by PCMCI+.

We used a standard approach to tokenization, removing punctuation, digits, and special characters. The text corpus was preprocessed by removing stopwords in English and Spanish from the NLTK Python library [52]. The terms were weighted using Term Frequency (TF). The number of topics was set to eight based on the balance between perplexity and UMass coherence scores (see S1 Fig in Supporting information). The LDA model was trained using the Gensim library [53] with a total of 30 passes, allowing for the model to stabilize. We used the default parameters for document-topic and topic-word distribution smoothing.

The main input parameter of the LDA algorithm is the number of topics. Following standard procedures, we plotted UMass coherence score values [54] as a function of the number of topics, and used the elbow of the curve (see S1 Fig in Supporting information) to identify the optimal number of topics [55,56]. Eight topics provided a relatively low coherence score of -3.6 (in the range of -14 to 14), and the number of topics was large enough to show a diverse set of discussions. We created mean topic proportion values per party, aggregating the average distribution of topics within tweets from members of each party.

## 3 Results

### 3.1 Effect of mass shooting lethality on gun-related discourse of legislators

The outcomes of all the logistic regressions (Models 1, 2, and 3) are summarized in Table 2. For Model 1, all independent variables are statistically significant, except for the residential segregation and race/ethnicity. Thus, we observed that the more fatalities, the higher the probability of members of the US Congress posting gun-related tweets. Likewise, the location control variable is significant, hinting that member of Congress reacts to mass shootings if they happen in the state the member represents. Finally, the Gini coefficient is also significant, suggesting that income inequality plays a role in the politician’s decision to make a public statement about the shooting. For Models 2 and 3, the lagged dependent variable in the autoregressive model is statistically significant, suggesting that past gun-related tweets are a predictor of current gun-related tweets. The lagged variable of the number of fatalities is also statistically significant, highlighting the connection between the incidents and the next day’s discussion of them by the members of Congress on X. Income inequality variable remains statistically significant for Model 3, but not for Model 2, and residential segregation index is not statistically significant across all models. Race/ethnicity is not statistically significant as a predictor of gun-related tweets across all models.

**Table 2:** Predictors of gun-related tweets by members of the US Congress. Odds ratios (ORs) with 95% confidence intervals (CIs) are presented. Results are based on mixed-effects logistic regression models with random intercepts for each member of Congress. Predictors are considered statistically significant if their *p* value is <0.050. Model 1 includes contemporaneous variables using the number of fatalities; Model 2 includes both contemporaneous and lagged variables using the number of fatalities; Model 3 includes both contemporaneous and lagged variables using total fatalities across all incidents. Location variable indicates whether the most lethal shooting event on the day occurred in the member’s state. Political affiliation is coded as one for Democrats and zero for Republicans. We additionally controlled for the race/ethnicity of the Congress members across all models.

	Model 1		Model 2		Model 3	
Variable	OR (95% CI)	*p* value	OR (95% CI)	*p* value	OR (95% CI)	*p* value
Constant	0.02 (0.01, 0.06)	0.000	0.01 (0.00, 0.02)	0.000	0.02 (0.01, 0.06)	0.000
Number of fatalities	1.10 (1.09, 1.11)	0.000	1.13 (1.12, 1.15)	0.000	–	–
Total number of fatalities	–	–	–	–	1.04 (1.03, 1.05)	0.000
Location	1.20 (1.07, 1.35)	0.003	1.24 (1.10, 1.41)	0.000	1.19 (1.05, 1.34)	0.006
Political affiliation	4.52 (3.72, 5.49)	0.000	3.60 (3.03, 4.28)	0.000	4.09 (3.41, 4.91)	0.000
Income inequality (Gini coefficient)	0.06 (0.01, 0.33)	0.001	0.20 (0.02, 1.72)	0.143	0.07 (0.01, 0.56)	0.012
Residential segregation index	1.00 (0.99, 1.00)	0.275	1.00 (1.00, 1.01)	0.855	1.00 (0.99, 1.00)	0.121
Previous day gun-related tweets	–	–	4.47 (4.12, 4.85)	0.000	4.43 (4.09, 4.80)	0.000
Previous day number of fatalities	–	–	1.33 (1.32, 1.35)	0.000	–	–
Previous day total number of fatalities	–	–	–	–	1.09 (1.08, 1.10)	0.000
Previous day location	–	–	0.74 (0.62, 0.89)	0.001	0.75 (0.63, 0.89)	0.001
**Race/ethnicity**						
Asian American	0.88 (0.54, 1.41)	0.585	0.90 (0.59, 1.37)	0.640	0.88 (0.57, 1.37)	0.576
Black	1.09 (0.81, 1.47)	0.567	1.10 (0.84, 1.43)	0.462	1.09 (0.82, 1.43)	0.555
Hispanic	0.79 (0.57, 1.10)	0.164	0.81 (0.61, 1.08)	0.169	0.81 (0.60, 1.10)	0.184
Multiracial	1.06 (0.48, 2.35)	0.882	1.04 (0.52, 2.09)	0.904	1.08 (0.52, 2.25)	0.841
Native American	0.61 (0.22, 1.72)	0.347	0.60 (0.23, 1.53)	0.292	0.62 (0.23, 1.64)	0.332
Akaike information criterion (AIC)	46777.7	–	44019.7	–	45739.2	–
Bayesian information criterion (BIC)	46849.8	–	44122.7	–	45893.7	–

**Fig 3:**
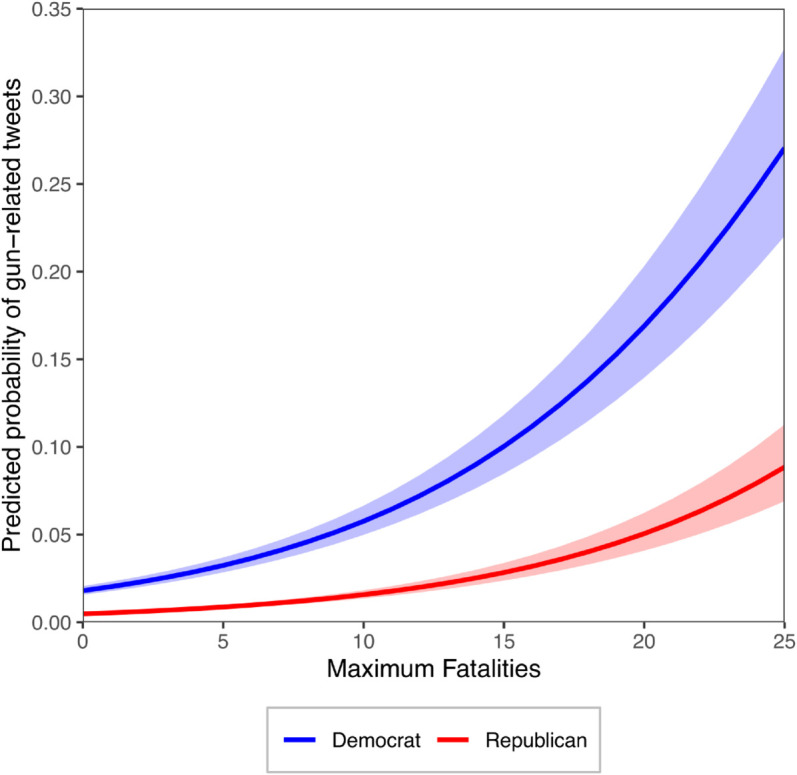
Effect of lethality on the response of the US Congress. Predicted probability of members of Congress responding to mass shootings on X as a function of the number of fatalities, based on Model 2. The shaded areas are the 95% confidence intervals. All other independent variables are held constant at their corresponding mean values.

Model 1 yielded an AIC of 46777.7 and a BIC of 46849.8. In comparison, the autoregressive models showed a substantially lower AIC (Model 2: 44019.7; Model 3: 45739.2) and BIC (Model 2: 44122.7; Model 3: 45893.7), suggesting that the autoregressive models, specifically Model 2, have a better fit for the data [45]. Fig 3 shows the predicted probabilities of a gun-related tweet being posted as a function of the number of fatalities, with separate curves for Democrats and Republicans (holding other variables constant). This analysis was conducted using the autoregressive Model 2 based on its higher accuracy. We notice that the more lethal a shooting was, the more likely members of Congress were to tweet about guns. This tendency was higher for Democrats than Republicans.

### 3.2 Identifying causal links between mass shootings and Congressional X activity

**Fig 4:**
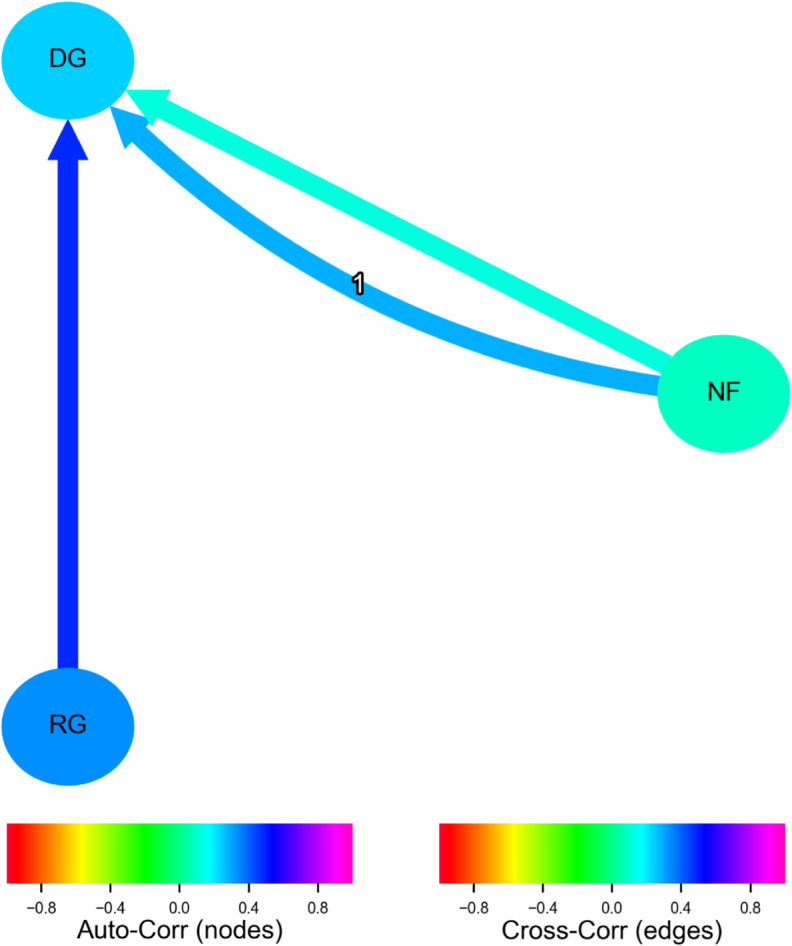
Discovered causal graph using the PCMCI+ algorithm for gun-related discourse by the US Congress. NF - number of fatalities of a single mass shooting per day, DG - proportion of Democrats’ tweeting about guns, RG - proportion of Republicans’ tweeting about guns. All relationships are statistically significant and contemporaneous, with NF also having a causal connection with DG at a time lag of one day. Colors represent partial correlation coefficients for links and autocorrelation coefficients for nodes.

Results of the PCMCI+ algorithm point at a significant causal link between the fatalities and the proportion of gun-related tweets among the Democrats in Congress (Fig 4). Specifically, a higher number of fatalities was positively associated with the probability of gun-related tweets among Democrats (partial correlation coefficient, *ρ*=0.122, *p*=0.001). This relationship is contemporaneous and at a time lag of one day, so that the observed causality should be interpreted in both Granger [57] and Pearl [48] senses. Democratic gun-related tweets were also thus significantly influenced by Republican gun-related tweets at lag 0 (*ρ*=0.483, *p*<0.001).

### 3.3 Substantive differences in Congressional responses to mass shootings by party

We found that the contents of those gun-related tweets posted in the aftermath of a mass shooting had significant differences across party lines. Republicans were overrepresented in topics of regulation and rights (mean topic proportion of 0.306 for Republicans versus 0.065 for Democrats), while Democrats tweeted more with messages centering on legislation (mean topic proportion of 0.349 for Democrats versus 0.097 for Republicans), victims, and gun violence itself (mean topic proportion of 0.257 for Democrats versus 0.075 for Republicans), as can be seen in Table 3.

**Table 3:** Mean topic proportions by political party of the US Congress members. Mean topic proportions derived from the LDA model, comparing the discourse of two political groups: Republicans and Democrats. These proportions indicate the relative frequency of each topic within the communications of each party. The top words for each topic are extracted from the model’s topic-word distribution, highlighting the most prominent terms associated with each topic.

Topic	Top words	Rep.	Dem.
Topic 1	violence, gun, health, survivors, public, crisis, community, prevention, mental, support	0.068	0.099
Topic 2	children, death, leading, cause, guns, joining, among, access, tune, firearms	0.047	0.032
Topic 3	gun, violence, communities, legislation, safety, act, lives, senate, pass, save	0.097	0.349
Topic 4	guns, background, gun, checks, weapons, hands, assault, firearms, ban, act	0.132	0.127
Topic 5	gun, rights, amendment, control, guns, democrats, second, citizens, americans, protect	0.306	0.065
Topic 6	court, laws, federal, supreme, right, risk, state, new, firearms, arms	0.099	0.036
Topic 7	gun, violence, must, lives, shooting, mass, lost, families, today, victims	0.075	0.257
Topic 8	biden, gun, atf, president, owners, rights, chipman, amendment, administration, David	0.175	0.034

## 4 Discussion

Over 1,300 mass shooting incidents were recorded by the Gun Violence Archive during a single US Congress. A proportion of these shootings makes the news, for a legislator to comment upon in a statement or a tweet. In our study, we set out to address three questions - does the fatality of a shooting affect the likelihood of a gun-related X post by a member of Congress? Is there a party difference? If so, what does that look like in terms of the issues raised when talking about guns?

Our findings indicate that the number of fatalities significantly influences the likelihood of members of Congress posting about guns. As the fatality count rises, the event increasingly captures the Congress’s attention. Within the attention-cycle theory [2], one may propose the existence of an issue-attention cycle which starts with heightened interest and then fades away. The interest peaks due to a “focusing” event [58], at which point the need for a solution from the policymakers is demanded by the public. Mass shootings seem to fit this pattern. This paper finds, however, that the cycle for mass shootings may be as short as two days, after which the issue of guns is not likely to be brought up in the X discourse of the members of the US Congress. It is tenable that this is an effect of the general desensitization in online communication towards violence that the country is experiencing [59].

For both parties, we registered a strong autocorrelation, suggesting a positive feedback effect wherein discussions within a party stimulate further discussions within the same party. A particularly interesting finding is the contemporaneous link between the proportion of gun-related tweets by Republicans and Democrats (controlled for fatalities of the mass shootings through partial correlation in the PCMCI+ implementation). The direction of the link (from Republicans to Democrats) highlights an interactive aspect of X discourse, where discourse within one party might be influenced by or responsive to the discourse within the other party. External events like mass shootings can, directly and indirectly, catalyze discussions and interactions between different political groups.

We found that as state-level income inequality increased, the members of Congress were more likely to post about guns in the aftermath of a mass shooting. While the adverse effect of income inequality on firearm violent crime has been known for decades [60], the connection between state-level income inequality and politicians’ social media response to a shooting is a new finding of this study. It is tenable that such a link rests upon politicians’ immediate framing of a shooting as the result of failed policy, health inequality, poverty, or other societal factors that are associated with violent crime and are valued by voters. Likewise, when mass shootings happened in a member’s home state, the members were also more likely to mention guns in their X messages. This supports the broader idea that members of Congress exhibit high responsiveness to their constituencies in social media communication, with healthcare high on the list of the communication agendas for both Democrats and Republicans [61]. Our findings do not point at race/ethnicity as a salient predictor of gun-related tweets by members of the Congress. Perhaps, this could be related to findings by Crifasi et al. [62] that “[w]hile support differed by race and ethnicity for some policies, majorities of U.S. adults supported nearly all gun policies examine regardless of race”. Overall, these findings are in line with the issue-attention cycle theory, whereby legislators feel compelled to comment on gun violence when it offers an entry point to gain political capital, appealing to broad societal issues, or when it impacts their constituency directly.

Our findings also indicate that Democrats are more likely to post about gun-related issues and that there is an association between a mass shooting and Democrats reacting to it via a post on X. Democrats, as the findings suggest, are responding to Republicans, as well as to the event itself. This may explain the discernible difference in the nature of the messaging that follows a mass shooting and support the claim that party members stick to the talking points or party line once a dramatic event calls out for a public reaction. Moreover, the observed responsiveness of Democrats to the Republican gun-related rhetoric may indicate that Democrats are also ‘activated’ by their opponent’s discourse on guns.

Not only do the dynamics of the responses differ between parties, but so does the content of the texts, highlighting the different framing used in response to mass shootings. For example, topics 5, 6, and 8 in Table 3 were disproportionately favored by the Republicans and centered on the issues of Second Amendment rights and regulations (via mentions of the ATF - Bureau of Alcohol, Tobacco, Firearms and Explosives). On the other hand, topics 3 and 7 were mostly invoked by the Democrats and revolved around issues of community, families, and victims, legislation, as well as the term ‘mass shooting’ itself. This can be explained as a product of political polarization, as well as of differing ways of framing the core issues [63]. This divergence may hinder bipartisan legislative efforts and the ability of Congress to showcase “institutional leadership” [64]. Interestingly, the very same difference in the issues chosen by members of Congress was also at the core of the political rhetoric of the recent Presidential candidates when discussing issues around gun violence in their political debate and in other public events. While Vice President Kamala Harris focused on victims and communities, President-elect Donald Trump stressed issues around crime and Second Amendment rights [65].

Our findings have important practical implications for policy and advocacy on the issue of gun violence. From the policy-making perspective, the transient nature of heightened attention suggests a narrow window of opportunity for policy intervention. Seizing these windows is challenged by dramatic differences in how the issue of gun violence is framed by policymakers. Appreciating commonalities and discrepancies between seemingly contrasting narratives may help policymakers reach out across the aisle and foster bipartisan discussion about gun policies. From the perspective of advocacy and public engagement, it is important to recognize the lifecycle of attention to the issue of gun violence and to develop measures that may prolong this attention beyond the immediate aftermath of tragic shootings. Sustained public interest and pressure on legislators may lead to a more systematic approach to addressing gun violence.

Our approach has several limitations. There are many more variables that play into an individual’s reaction to a mass shooting, all of which we cannot account for. The proposed model provides quantitative and qualitative insight into the effect of some of the most basic aspects we know about a shooting - how many people were killed or where the shooting took place - on the public response by members of Congress. We focused only on one Congress, encompassing its entire two-year term. Although a substantial proportion of members stay in office for more than one term, the results of such a study will always vary with changes in Congress, which can be explored in future research.

The effects of mass shootings on public discourse are well studied [27,66,67], from the general public’s interest [25] to media coverage [26], but much less is known about the response of policymakers to these tragic events. Despite its limitations, our study provides valuable insight into the interplay of mass shootings and political communication. Our analysis identified an association between mass shootings’ severity and the reaction by Democrats via gun-related tweets in the immediate aftermath and on the day after the shooting. We also discovered a link between the discourses of Republicans and Democrats on the issue. The substance of the tweets was found to differ based on the party affiliation of the member of Congress, with Democrats focusing on mass shooting itself as a term, violence, community, family, and legislature, and the Republican tweets associated with topics on law enforcement, crime, and Second Amendment rights.

### Consent for publication

The data for this study were obtained from publicly accessible sources, and no personally identifiable information was utilized. Therefore, explicit consent for publication is not required.

## Supporting information

S1 Fig UMass coherence score versus the number of topics. Eight topics were selected as a trade-off between the coherence score and a diverse set of topics.
(EPS)
